# Cancer mortality predictions for 2021 in Latin America

**DOI:** 10.1097/CEJ.0000000000000702

**Published:** 2021-07-08

**Authors:** Greta Carioli, Paola Bertuccio, Matteo Malvezzi, Paolo Boffetta, Fabio Levi, Eva Negri, Carlo La Vecchia

**Affiliations:** a Department of Clinical Sciences and Community Health; b Department of Biomedical and Clinical Sciences L. Sacco, Università degli Studi di Milano, Milan, Italy; c Stony Brook Cancer Center, Stony Brook University, Stony Brook, New York, USA; d Department of Medical and Surgical Sciences, University of Bologna, Bologna, Italy; e Department of Epidemiology and Health Services Research, Center for Primary Care and Public Health (Unisantè), University of Lausanne, Lausanne, Switzerland; f Department of Humanities, Pegaso Online University, Naples, Italy

**Keywords:** cancer, colorectal cancer, Latin America, mortality, projections

## Abstract

We estimated cancer mortality statistics for the current year in seven major Latin American countries, with a focus on colorectal cancer. We retrieved official death certification data and population figures from the World Health Organization and the Pan American Health Organization databases. We analysed mortality from all neoplasms combined and for selected cancer sites. We estimated numbers of deaths and age-standardized mortality rates for the year 2021 using a logarithmic Poisson count data joinpoint model. Total cancer mortality is predicted to decline in all countries considered for both sexes, with the exception of Argentinian women. The lowest total mortality rates were predicted in Mexico (65.4/100 000 men and 62.3 in women), the highest ones were in Cuba (133.3/100 000 men and 91.0 in women). Stomach cancer rates have been decreasing since 1970 in all countries; colorectal cancer started to decline over recent calendar periods. Rates for this cancer were unfavourable in the youngest age group. Lung cancer trends declined in males and remained comparatively low in all countries except Cuba. In Cuba, lung cancer rates in women overtook those for breast. Mortality from cancers of the breast, (cervix) uterus, ovary, prostate and bladder, as well as leukemia mostly showed favourable trends. A marked variability in rates across Latin American countries persists, and rates were relatively high for stomach, uterus, prostate and lung cancers, as compared to Europe and North America, suggesting the need to improve preventive strategies. Colorectal cancer mortality was relatively low in Latin America, except in Argentina, and short-term predictions remain moderately favourable.

## Introduction

Official cancer mortality figures are important for decisions in public health and for resource allocation; however, they are available with a delay of some years. Since 2011, we predicted numbers of cancer deaths and corresponding rates for the European Union (EU) and its most populous countries (Carioli *et al.*, 2021), and since 2017, for other areas worldwide, including Latin America and Australasia, Russia and Ukraine (Carioli *et al.*, 2020; Pizzato *et al.*, 2021). Similar predictions have also long been published in the USA (Siegel *et al.*, 2021). Those analyses on different geographical areas highlighted declines in cancer mortality rates, but not in absolute number of deaths, in most countries over the last decades.

In our previous work on Latin America (Carioli *et al.*, 2020), we found overall lower cancer rates as compared to other areas worldwide, except for stomach and (cervix) uterus cancer, which remained exceedingly high. Predicted cancer trends were less favourable in women from Argentina (particularly, for all neoplasms and lung cancer) and Cuba (stomach and breast cancer). Here, we update cancer death certification data for seven major Latin America countries, and we predict figures for 2021 applying the same methodology of previous works.

## Materials and methods

We extracted official deaths certification data for all neoplasms combined and 10 major cancer sites, that is, stomach, colorectum, pancreas, lung, breast, uterus (cervix and corpus), ovary, prostate, bladder cancer and leukaemia, from the WHO database (World Health Organization Statistical Information System). We re-coded cancer deaths according to the 10th Revision of the International Classification of Diseases (ICD) (World Health Organization, 1992). We obtained data for Latin American countries with over 10 million inhabitants and over 85% deaths certification coverage (Mathers *et al.*, 2005). Thus, we included Argentina, Brazil, Chile, Colombia, Cuba, Mexico and Venezuela. We considered the period between 1970 and 2017 (2014 for Venezuela). We retrieved resident population estimates, based on official censuses, from the Pan American Health Organization (PAHO) database (Pan American Health Organization, 2019).

Using the matrices of certified deaths and resident populations, we derived age-specific mortality rates for each 5-year age group (from 0–4 to 80+ years), sex, calendar year and cancer site. We standardized mortality rates by 5-year age groups using the world standard population for all ages and, for colorectal cancer, we also calculated rates for the 30–49, 50–69 and 70+ age groups.

We fit a Poisson joinpoint regression model (Kim *et al.*, 2000) to the number of deaths (log scale) in each 5-year age group to predict mortality figures for 2021. This model identified significant changes in trends testing from 0 to 5 joinpoints. To estimate age-specific numbers of deaths for 2021 and the corresponding 95% prediction intervals (PIs), we fit a linear regression model to the mortality data for each age group over the most recent trend segment identified by the joinpoint model. Using predicted age-specific death counts and predicted populations from the PAHO database (Pan American Health Organization, 2019), we computed age-specific and age-standardized (world standard population) death rates for 2021 and their 95% PIs.

We estimated numbers of averted cancer deaths for all neoplasms over the years 1991–2021 by comparing observed and expected deaths on the basis of the 1990 age-specific rates, since most selected countries showed the highest rates around that year.

## Data availability statement

The data that support the findings of this study are openly available in the WHO database at http://www.who.int/healthinfo/statistics/mortality_rawdata/en/index.html, reference number (World Health Organization Statistical Information System, 2019).

## Results

**Fig. 1:**
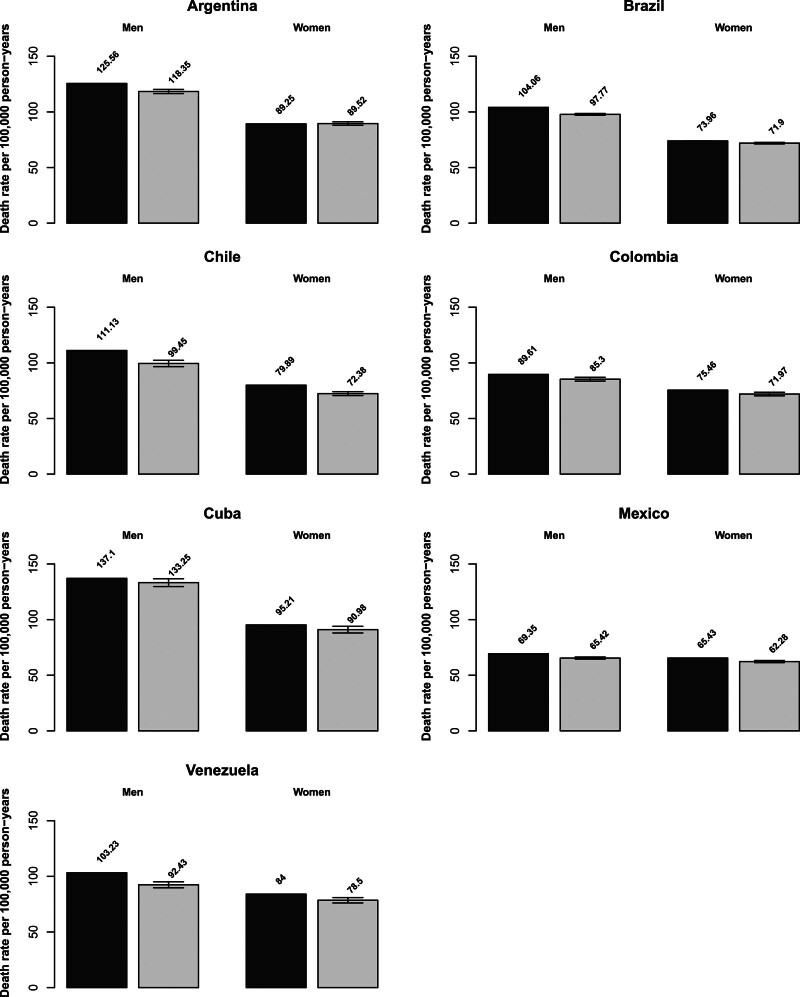
Bar-plots of age-standardized (world population) death rates per 100 000 persons for the 2015–2017 triennium (dark grey) and predicted rates for 2021 (light grey), with 95% prediction intervals (PIs) for total cancers in the seven selected Latin American countries, men and women.

Tables 1 (men) and 2 (women) include the number of predicted cancer deaths and rates for the year 2021 with the corresponding 95% PIs along with data observed for the 2015–2017 triennium, and the percent differences between 2021 and 2015–2017 rates for the seven countries and the 10 cancer sites considered. Figure 1 shows bar plots of age-standardized death rates per 100 000 population from all neoplasms in the countries considered, in men and women, in the 2015–2017 triennium (dark grey), and the predicted rates for 2021 (light grey), with corresponding 95% PIs.

**Table 1:** Numbers of predicted deaths and mortality rates per 100 000 men for the year 2021 and comparison figures for the 2015–2017 triennium (2014 only for Venezuela), from the seven selected Latin American countries, with 95% prediction intervals and percent differences between 2021 and 2015–2017 rates

		Observed number of deaths 2015–2017^a^	Predicted number of deaths 2021 (95% PI)	Observed ASR^b^ 2015–2017^a^	Predicted ASR^b^ 2021 (95% PI)	% difference 2020 vs. 2015–2017
Argentina	Stomach	1901	1980 (1892–2073)	7.18	6.83 (6.53–7.14)	−4.8
	Colorectum	4296	4620 (4451–4797)	15.73	15.32 (14.72–15.92)	−2.6
	Pancreas	1998	2190 (2089–2298)	7.48	7.4 (7.04–7.76)	−1.1
	Lung	6384	6250 (5996–6499)	24.4	21.29 (20.38–22.2)	−12.8
	Prostate	3790	3650 (3469–3832)	12.2	10.59 (10.08–11.1)	−13.2
	Bladder	1075	1070 (990–1145)	3.71	3.32 (3.07–3.57)	−10.5
	Leukaemias	1046	1110 (1043–1180)	4.13	4.07 (3.8–4.34)	−1.5
	All cancers	33 806	35 260 (34 684–35 843)	125.56	118.35 (116.52–120.18)	−5.7
Brazil	Stomach	9253	9740 (9514–9972)	8.43	7.39 (7.21–7.56)	−12.4
	Colorectum	11 218	13 190 (12 909–13 470)	10.18	9.95 (9.73–10.18)	−2.2
	Pancreas	4991	5910 (5707–6105)	4.58	4.53 (4.37–4.69)	−1.1
	Lung	15 869	17 160 (16 851–17 477)	14.6	13 (12.76–13.24)	−11
	Prostate	14 933	16 840 (16 508–17 163)	13.25	12.01 (11.75–12.26)	−9.4
	Bladder	2814	3000 (2835–3172)	2.52	2.18 (2.06–2.3)	−13.4
	Leukaemias	3711	3930 (3785–4071)	3.47	3.22 (3.1–3.35)	−7.1
	All cancers	114 214	128 470 (127 394–129 548)	104.06	97.77 (96.94–98.61)	−6
Chile	Stomach	2197	2230 (2112–2354)	17.27	14.57 (13.72–15.42)	−15.6
	Colorectum	1341	1470 (1394–1551)	10.49	9.66 (9.14–10.17)	−7.9
	Pancreas	650	740 (693–791)	5.21	4.99 (4.64–5.34)	-4.2
	Lung	1872	1920 (1786–2046)	15.08	13.06 (12.21–13.9)	−13.4
	Prostate	2130	2280 (2163–2391)	14.6	12.78 (12.16–13.41)	−12.4
	Bladder	368	350 (313–392)	2.71	2.16 (1.92–2.4)	−20.2
	Leukaemias	416	400 (362–446)	3.62	3.18 (2.8–3.56)	−12.2
	All cancers	14 227	15 300 (14 900–15 697)	111.13	99.45 (96.63–102.27)	−10.5
Colombia	Stomach	3150	3380 (3254–3499)	12.94	11.46 (11.03–11.88)	−11.5
	Colorectum	1885	1900 (1760–2045)	7.73	6.42 (5.94–6.89)	−17
	Pancreas	858	980 (905–1055)	3.56	3.35 (3.08–3.61)	−6
	Lung	2718	2910 (2774–3044)	11.39	9.77 (9.32–10.23)	−14.2
	Prostate	2969	3290 (3141–3435)	12.08	10.65 (10.17–11.12)	−11.9
	Bladder	341	350 (314–386)	1.4	1.16 (1.04–1.28)	−16.9
	Leukaemias	1030	1020 (951–1087)	4.28	3.81 (3.54–4.07)	−11.1
	All cancers	21 750	25 060 (24 553–25 570)	89.61	85.3 (83.61–86.99)	−4.8
Cuba	Stomach	547	570 (527–617)	5.19	4.84 (4.4–5.28)	−6.7
	Colorectum	1202	1290 (1216–1369)	10.74	10.31 (9.62–10.99)	−4
	Pancreas	439	480 (429–521)	4.23	4.14 (3.69–4.59)	−2.1
	Lung	3551	3760 (3570–3948)	33.97	31.45 (29.84–33.06)	−7.4
	Prostate	3042	3190 (3001–3376)	23.24	20.97 (19.89–22.06)	−9.8
	Bladder	483	520 (475–569)	4.09	3.91 (3.53–4.29)	−4.4
	Leukaemias	333	320 (284–356)	3.84	3.25 (2.65–3.85)	−15.4
	All cancers	14 707	16 390 (15 956–16 832)	137.1	133.25 (129.72–136.78)	−2.8
Mexico	Stomach	3266	3430 (3301–3550)	5.42	4.8 (4.62–4.97)	−11.5
	Colorectum	3491	3660 (3465–3845)	5.82	5.15 (4.89–5.41)	−11.5
	Pancreas	2001	2210 (2103–2309)	3.41	3.13 (2.98–3.28)	−8.3
	Lung	4241	4330 (4165–4488)	7.08	6 (5.77–6.24)	−15.2
	Prostate	6529	7160 (6909–7412)	10.02	8.94 (8.65–9.24)	−10.8
	Bladder	782	770 (717–833)	1.26	1.02 (0.95–1.1)	−18.7
	Leukaemias	2384	2480 (2380–2584)	3.77	3.55 (3.4–3.7)	−5.8
	All cancers	42 359	47 520 (46 834–48 211)	69.35	65.42 (64.51–66.33)	−5.7
Venezuela	Stomach	1113	1260 (1173–1348)	8.54	7.47 (6.95–7.98)	−12.5
	Colorectum	999	1070 (1009–1139)	7.7	6.41 (6.02–6.79)	−16.8
	Pancreas	488	530 (483–580)	3.83	3.2 (2.9–3.5)	−16.6
	Lung	2151	2190 (2032–2345)	16.88	13.16 (12.25–14.07)	−22.1
	Prostate	2585	3020 (2882–3162)	20.5	17.81 (16.99–18.62)	−13.1
	Bladder	209	210 (181–235)	1.63	1.23 (1.07–1.39)	−24.4
	Leukaemias	481	530 (483–573)	3.38	3.18 (2.91–3.46)	−5.8
	All cancers	13 416	15 470 (14 996–15 949)	103.23	92.43 (89.75–95.11)	−10.5

PI, prediction interval.

^a^2014 for Venezuela.

^b^ASR, age-standardized mortality rates using the world standard population.

**Table 2:** Numbers of predicted deaths and mortality rates per 100 000 women for the year 2021 and comparison figures for the 2015–2017 triennium (2014 only for Venezuela), from seven selected Latin American countries, with 95% prediction intervals and percent differences between 2021 and 2015–2017 rates

		Observed number of deaths 2015–2017^a^	Predicted number of deaths 2021 (95% PI)	Observed ASR^b^ 2015–2017^a^	Predicted ASR^a^ 2021 (95% PI)	% difference 2020 vs. 2015–2017
Argentina	Stomach	1068	1130 (1051–1207)	2.93	2.87 (2.67–3.08)	−2
	Colorectum	3687	3760 (3580–3948)	9.44	9.02 (8.66–9.38)	−4.4
	Pancreas	2230	2360 (2251–2465)	5.67	5.46 (5.18–5.74)	−3.7
	Lung	3137	3420 (3271–3566)	9.32	9.36 (8.94–9.78)	0.4
	Breast	5924	6320 (6029–6609)	17.5	16.77 (16.02–17.53)	−4.2
	Uterus (cervix and corpus)	2830	3020 (2902–3144)	9.56	9.45 (9.02–9.87)	−1.2
	Ovary	1221	1260 (1183–1340)	3.83	3.63 (3.4–3.87)	−5.1
	Bladder	343	390 (345–434)	0.78	0.8 (0.72–0.89)	3.2
	Leukaemias	843	840 (772–913)	2.63	2.44 (2.22–2.65)	−7.3
	All cancers	31 229	33 740 (32 709–34 780)	89.25	89.52 (87.93–91.11)	0.3
Brazil	Stomach	5155	5370 (5166–5565)	3.64	3.25 (3.12–3.37)	−10.8
	Colorectum	11 521	13 600 (13 282–13 912)	8.06	7.99 (7.8–8.18)	−0.9
	Pancreas	5051	5780 (5600–5951)	3.51	3.31 (3.21–3.42)	−5.6
	Lung	11 355	13 110 (12 694–13 530)	8.28	7.93 (7.65–8.22)	−4.2
	Breast	16 065	18 160 (17 762–18 558)	11.95	11.63 (11.35–11.91)	−2.6
	Uterus (cervix and corpus)	9739	10 960 (10 583–11 340)	7.3	7.19 (6.92–7.45)	−1.6
	Ovary	3775	4220 (4066–4374)	2.84	2.71 (2.61–2.81)	−4.6
	Bladder	1272	1270 (1173–1369)	0.84	0.69 (0.64–0.74)	−17.6
	Leukaemias	3184	3400 (3251–3541)	2.49	2.33 (2.22–2.44)	−6.4
	All cancers	101 703	116 570 (115 553–117 592)	73.96	71.9 (71.23–72.56)	−2.8
Chile	Stomach	1109	1160 (1073–1241)	6.33	5.67 (5.28–6.07)	−10.4
	Colorectum	1396	1530 (1444–1624)	7.87	7.46 (7.07–7.86)	−5.2
	Pancreas	780	870 (816–925)	4.59	4.36 (4.07–4.64)	−5.1
	Lung	1311	1270 (1156–1379)	7.91	6.59 (6.09–7.08)	−16.7
	Breast	1502	1520 (1443–1596)	10.01	8.66 (8.2–9.12)	−13.5
	Uterus (cervix and corpus)	951	910 (841–970)	6.57	5.43 (4.99–5.88)	−17.3
	Ovary	486	510 (460–554)	3.4	3.12 (2.8–3.44)	−8.3
	Bladder	181	190 (166–214)	0.97	0.83 (0.71–0.95)	−14.6
	Leukaemias	363	360 (323–397)	2.64	2.34 (2.03–2.65)	−11.5
	All cancers	12 971	13 850 (13 509–14 181)	79.89	72.38 (70.64–74.13)	−9.4
Colombia	Stomach	1983	2210 (2096–2325)	6.47	6.05 (5.75–6.34)	−6.5
	Colorectum	2070	2100 (1947–2245)	6.78	5.76 (5.39–6.13)	−15
	Pancreas	959	1080 (1021–1131)	3.16	2.92 (2.77–3.07)	−7.5
	Lung	1978	2070 (1962–2169)	6.57	5.59 (5.32–5.86)	−14.9
	Breast	3107	3270 (3087–3462)	10.59	9.72 (9.2–10.25)	−8.2
	Uterus (cervix and corpus)	2291	2500 (2372–2622)	7.8	7.25 (6.86–7.63)	−7.1
	Ovary	1027	1110 (1040–1177)	3.54	3.25 (3.05–3.46)	−8.2
	Bladder	164	180 (151–206)	0.51	0.45 (0.38–0.52)	−10.9
	Leukaemias	866	880 (811–944)	3.15	2.88 (2.66–3.1)	−8.6
	All cancers	22511	25 360 (24 754–25 973)	75.46	71.97 (70.4–73.55)	−4.6
Cuba	Stomach	350	380 (339–413)	2.9	2.76 (2.42–3.1)	−4.8
	Colorectum	1575	1620 (1532–1708)	12.16	11.02 (10.43–11.61)	−9.4
	Pancreas	410	470 (423–515)	3.42	3.45 (3.08–3.82)	0.9
	Lung	2045	2180 (2074–2290)	18.32	17.07 (16.05–18.09)	−6.8
	Breast	1534	1650 (1543–1752)	13.61	13.17 (12.13–14.21)	−3.2
	Uterus (cervix and corpus)	1180	1190 (1106–1276)	11.13	9.91 (8.97–10.85)	−11
	Ovary	314	350 (308–389)	3.14	3.21 (2.77–3.64)	2.1
	Bladder	179	200 (175–219)	1.31	1.27 (1.1–1.43)	−3.3
	Leukaemias	252	230 (196–272)	2.78	2.22 (1.64–2.79)	−20.2
	All cancers	10720	11 580 (11 273–11 891)	95.21	90.98 (87.99–93.98)	−4.4
Mexico	Stomach	2833	2920 (2810–3034)	4.1	3.56 (3.42–3.7)	−13.2
	Colorectum	3111	3250 (3113–3383)	4.54	3.94 (3.76–4.11)	−13.3
	Pancreas	2208	2400 (2317–2488)	3.25	2.92 (2.81–3.04)	−10.1
	Lung	2607	2870 (2725–3008)	3.82	3.53 (3.34–3.72)	−7.5
	Breast	6490	7260 (7060–7470)	9.85	9.43 (9.16–9.69)	−4.3
	Uterus (cervix and corpus)	5021	5390 (5217–5559)	7.55	6.92 (6.69–7.16)	−8.3
	Ovary	2495	2430 (2267–2597)	3.84	3.13 (2.91–3.36)	−18.4
	Bladder	327	320 (261–374)	0.44	0.35 (0.3–0.41)	−19.9
	Leukaemias	2051	2090 (2000–2185)	3.12	2.87 (2.73–3)	−8.2
	All cancers	44127	49 500 (48 767–50 235)	65.43	62.28 (61.29–63.26)	−4.8
Venezuela	Stomach	726	810 (749–873)	4.49	3.92 (3.6–4.24)	−12.7
	Colorectum	998	1180 (1086–1281)	6.35	5.94 (5.43–6.45)	−6.4
	Pancreas	529	510 (468–552)	3.38	2.51 (2.3–2.71)	−25.9
	Lung	1457	1650 (1559–1735)	9.58	8.14 (7.7–8.59)	−15
	Breast	2204	2470 (2320–2615)	14.29	13.11 (12.34–13.87)	−8.3
	Uterus (cervix and corpus)	2065	2260 (2168–2357)	13.23	11.91 (11.39–12.42)	−10
	Ovary	503	520 (478–558)	3.31	2.72 (2.5–2.93)	−17.9
	Bladder	111	110 (95–129)	0.67	0.51 (0.42–0.59)	−24.3
	Leukaemias	409	440 (400–479)	2.61	2.41 (2.18–2.65)	−7.5
	All cancers	13111	15 260 (14 752–15 776)	84	78.5 (76.09–80.9)	−6.6

PI, prediction interval.

^a^2014 for Venezuela.

^b^ASR, age-standardized mortality rates using the world standard population.

In men, mortality from all neoplasms is predicted to decline in all countries. Falls in rates between 2015–2017 and 2021 ranged between −2.8% for Cuba and over −10% in Chile and Venezuela. Mexico showed the lowest rates, 69.4/100 000 men in 2015–2017 and 65.4 in 2021. The highest observed and predicted rates were in Cuba, 137.1 and 133.3, respectively. In women, except for Argentina where rates from all neoplasms remained stable (0.3% between 2015–2017 and 2021), total cancer mortality is predicted to decrease. Declines ranged between −2.8% in Brazil and −9.4% in Chile. Falls in women were however generally smaller than in men. Mexico had the lowest rates, 65.4/100 000 women in 2015–2017 and 62.3 in 2021 and Cuba showed the highest ones, 95.2/100 000 in 2015–2017 and 91.0 in 2021. Numbers of deaths are however predicted to rise in 2021 as compared to 2015–17, in all countries and both sexes; Venezuela showed the largest increases in absolute number of cancer deaths, over 15% since 2015–2017 in both sexes.

**Fig. 2:**
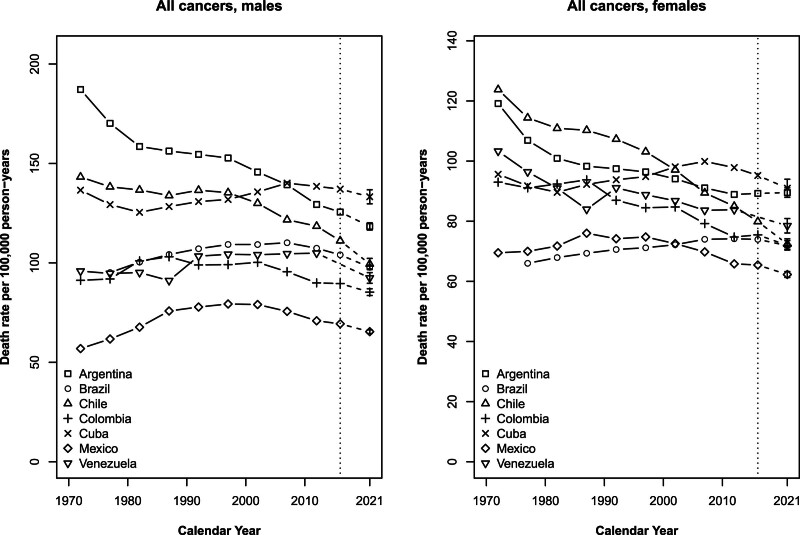
Age-standardized (world population) death rates from total cancers in quinquennia from 1970 to 2014 plus the 2015–2017 triennium and predicted rates for 2021, with 95% prediction intervals (PIs), for Argentina (squares), Brazil (circles), Chile (triangles), Colombia (crosses), Cuba (xs), Mexico (diamonds) and Venezuela (inverted triangles), in men and women.

Figure 2 shows trends in total cancer mortality rates, in men and women separately, from the 1970–1974 quinquennium to the 2015–2017 triennium, and predicted rates for 2021 with the corresponding PIs. Rates in Argentina decreased over the whole period in men. Trends for Chile, Colombia and Mexico started to decline between 1990 and 2000; Brazil, Cuba and Venezuela had declines in rates later over the last decade. In women, rates in Argentina, Chile, Colombia and Venezuela declined over the whole period, while mortality rates in Argentina only tended to level off over the last decade. Trends in Cuba and Mexico started to decrease and to stabilize in Brazil around 2000.

**Fig. 3:**
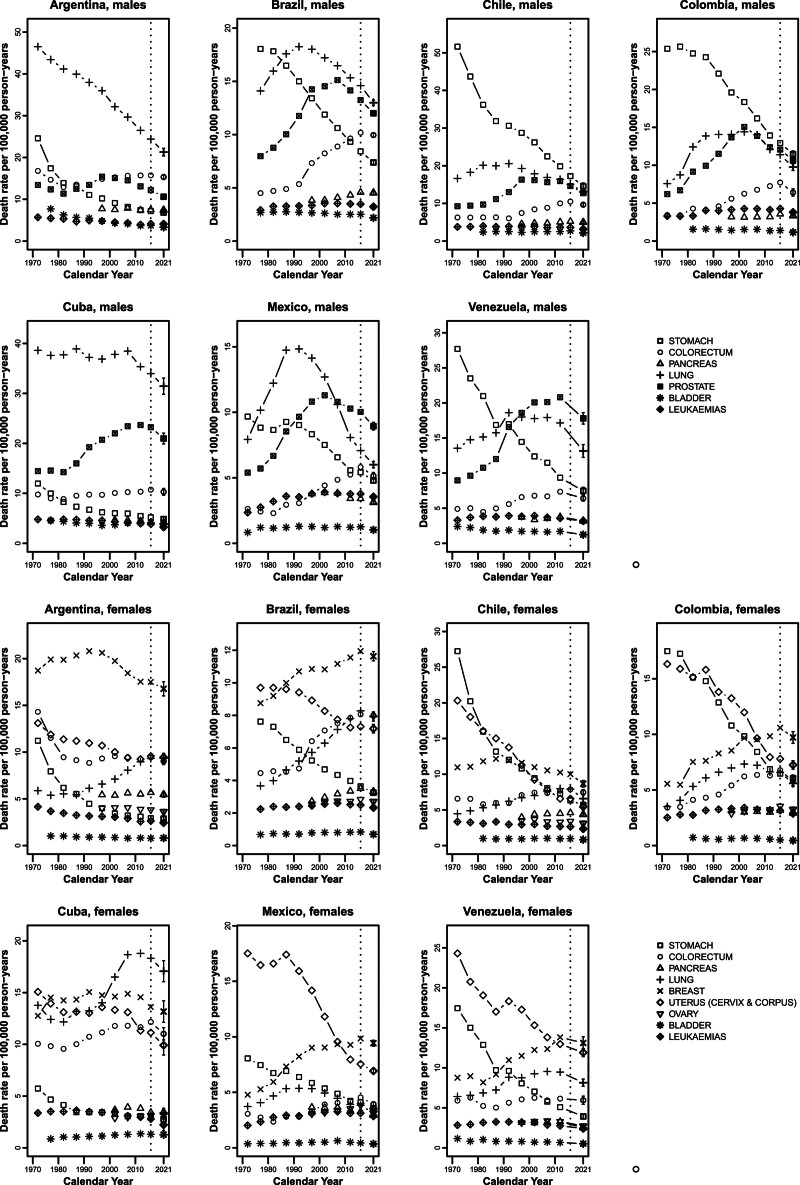
Age-standardized (world population) death rates from selected major cancers in quinquennia from 1970 to 2014 plus the 2015–2017 triennium and predicted rates for 2021, with 95% prediction intervals (PIs) for seven selected Latin American countries. Men: stomach (squares), colorectum (circles), pancreas (triangles), lung (crosses), prostate (ticked squares), bladder (asterisks) and leukaemias (ticked diamonds). Women: stomach (squares), colorectum (circles), pancreas (triangles), lung (crosses), breast (xs), uterus (cervix and corpus) (diamonds), ovary (inverted triangles), bladder (asterisks) and leukaemias (ticked diamonds).

Figure 3 gives mortality trends for each cancer site and country analysed. In men, stomach cancer rates have been falling since 1970 in all countries. Trends for colorectal cancer increased until the 1990s, and were more favourable in most recent years. In most countries, lung cancer rates started to decline around the 1990s (the 2000s for Colombia); Argentina showed favourable rates since the 1970s and Cuba only over the last decade, starting from comparatively high rates. The lung cancer predicted rate in Cuba was the highest in Latin America, with a value of 31.5/100 000 men. Mexico showed the lowest rate for 2021, 6.0. Prostate cancer rates have been rising since the early 2000s, and only declined over the last period, however maintaining relatively high rates. Bladder cancer and leukaemias showed modest declines in most countries, reaching predicted rates below 5/100 000 men. In women, stomach cancer rates were declining over the whole period. Colorectal cancer rates were favourable over the most recent years. Some falls were observed for pancreatic cancer in both sexes, too. In most countries, female lung cancer rates started to level off or decline over most recent periods (falls in Mexico started earlier compared to other countries), though after increases. Argentina had a stable predicted rate. Female lung cancer rates in Cuba, although favourable over the last decade, remained the highest (17.1/100 000 women in 2021) and were also higher than breast cancer rates (13.2). Breast cancer patterns were favourable, particularly in the most recent years. Uterine (including cervix) cancer rates were declining over the whole period in all countries, remaining, however, comparatively high. Ovarian, bladder cancer and leukaemias showed some declines, with values of predicted rates lower than 4/100 000 women.

Table 3 presents the age-standardized mortality rates at all ages, and at the age groups 30–49, 50–69 and 70+ for colorectal cancer in the 2012–2014 and 2015–2017 triennia, the predicted rates for 2021 and the percent difference between 2021 and 2015–2017 (2012–2014 for Venezuela). With few exceptions, that is, the youngest age groups in Brazil, Colombia, Cuba and Venezuela, men showed favourable patterns in all countries, with generally larger falls in those aged 70+. Rates are predicted to decrease in women too, for most countries and age groups; exceptions are the youngest age groups in Brazil, Chile and Cuba and those aged 50–69 in Argentina, Brazil and Venezuela.

**Table 3:** Age-standardized colorectal cancer mortality rates for all ages, 30–49, 50–69, 70+ years age groups, in men and women in seven selected Latin American countries

		Men				Women			
		Observed ASR^a^ 2012–2014	Observed ASR^a^ 2015–2017	Predicted ASR^a^ 2021 (95% PI)	% difference 2021 vs. 2015–2017	Observed ASR^a^ 2012–2014	Observed ASR^a^ 2015–2017	Predicted ASR^a^ 2021 (95% PI)	% difference 2021 vs. 2015–2017
Argentina	All ages	15.67	15.73	15.32 (14.72–15.92)	−2.6	9.32	9.44	9.02 (8.66–9.38)	−4.4
	Truncated 30–49 years	4.6	4.81	4.49 (3.98–5.01)	−6.6	4.4	4.32	4.17 (3.64–4.7)	−3.4
	Truncated 50–69 years	44.85	45.63	45.02 (41.94–48.11)	−1.3	26.47	27.96	28.44 (26.9–29.99)	1.7
	Truncated 70+ years	180.98	178.49	172.38 (164.43–180.32)	−3.4	98.27	95.74	84.18 (78.48–89.89)	−12.1
Brazil	All ages	9.69	10.18	9.95 (9.73–10.18)	−2.2	7.94	8.06	7.99 (7.8–8.18)	−0.9
	Truncated 30–49 years	3.55	3.95	3.99 (3.78–4.2)	1	3.93	3.99	4.01 (3.73–4.3)	0.6
	Truncated 50–69 years	27.75	29.2	29.21 (28.12–30.3)	0	22.24	23.02	23.66 (22.79–24.52)	2.8
	Truncated 70+ years	107.78	111.64	105.49 (102.37–108.61)	−5.5	84.09	83.72	78.67 (76–81.34)	−6
Chile	All ages	10.21	10.49	9.66 (9.14–10.17)	−7.9	7.83	7.87	7.46 (7.07–7.86)	−5.2
	Truncated 30–49 years	3.08	3.71	3.31 (2.72–3.89)	−10.9	3.11	3.28	3.49 (2.91–4.06)	6.3
	Truncated 50–69 years	27.2	27.32	25.39 (23.17–27.62)	−7.1	20.89	21.13	20.22 (18.48–21.97)	−4.3
	Truncated 70+ years	126.12	128.34	118.01 (109.57–126.45)	−8.1	92.88	91.68	83.52 (77.66–89.38)	−8.9
Colombia	All ages	7.37	7.73	6.42 (5.94–6.89)	−17	6.29	6.78	5.76 (5.39–6.13)	−15
	Truncated 30–49 years	2.68	2.8	2.85 (2.37–3.33)	1.8	2.82	3.06	3.03 (2.62–3.43)	−1
	Truncated 50–69 years	19.48	19.96	16.29 (14.57–18)	−18.4	15.78	17.36	15.35 (13.97–16.74)	−11.6
	Truncated 70+ years	87.01	93.44	74.42 (65.25–83.59)	−20.4	74.96	79.07	61.76 (54.8–68.73)	−21.9
Cuba	All ages	10.47	10.74	10.31 (9.62–10.99)	−4	11.57	12.16	11.02 (10.43–11.61)	−9.4
	Truncated 30–49 years	3.23	2.9	3.17 (1.83–4.52)	9.4	3.46	4.03	5.15 (4.01–6.28)	27.7
	Truncated 50–69 years	28.36	27.8	25.47 (22.88–28.07)	−8.4	30.48	33.19	27.12 (24.63–29.61)	−18.3
	Truncated 70+ years	126.8	136.44	131.45 (121.73–141.17)	−3.7	144.54	144.53	134.04 (125.81–142.27)	−7.3
Mexico	All ages	5.43	5.82	5.15 (4.89–5.41)	−11.5	4.29	4.54	3.94 (3.76–4.11)	−13.3
	Truncated 30–49 years	2.65	3.07	2.86 (2.56–3.16)	−6.9	2.44	2.51	2.37 (2.12–2.61)	−5.7
	Truncated 50–69 years	16.72	17.75	15.84 (14.64–17.05)	−10.8	12.92	13.79	11.07 (10.19–11.95)	−19.7
	Truncated 70+ years	50.75	54.14	46.23 (42.28–50.17)	−14.6	39.86	41.82	38.65 (36.61–40.7)	−7.6
Venezuela	All ages	7.27	.	6.41 (6.02–6.79)	−11.9	6.21		5.94 (5.43–6.45)	−4.3
	Truncated 30–49 years	2.96	.	3.17 (2.67–3.67)	7.2	2.64		2.56 (2.12–2.99)	−3.2
	Truncated 50–69 years	21.65	.	20.12 (18.49–21.75)	−7.1	18.52		20.29 (17.56–23.03)	9.6
	Truncated 70+ years	74.76	.	57.51 (51.1–63.93)	−23.1	63.12		49.61 (43.57–55.65)	−21.4

PI, prediction interval.

^a^ASR, age-standardized mortality rates using the world standard population.

**Fig. 4:**
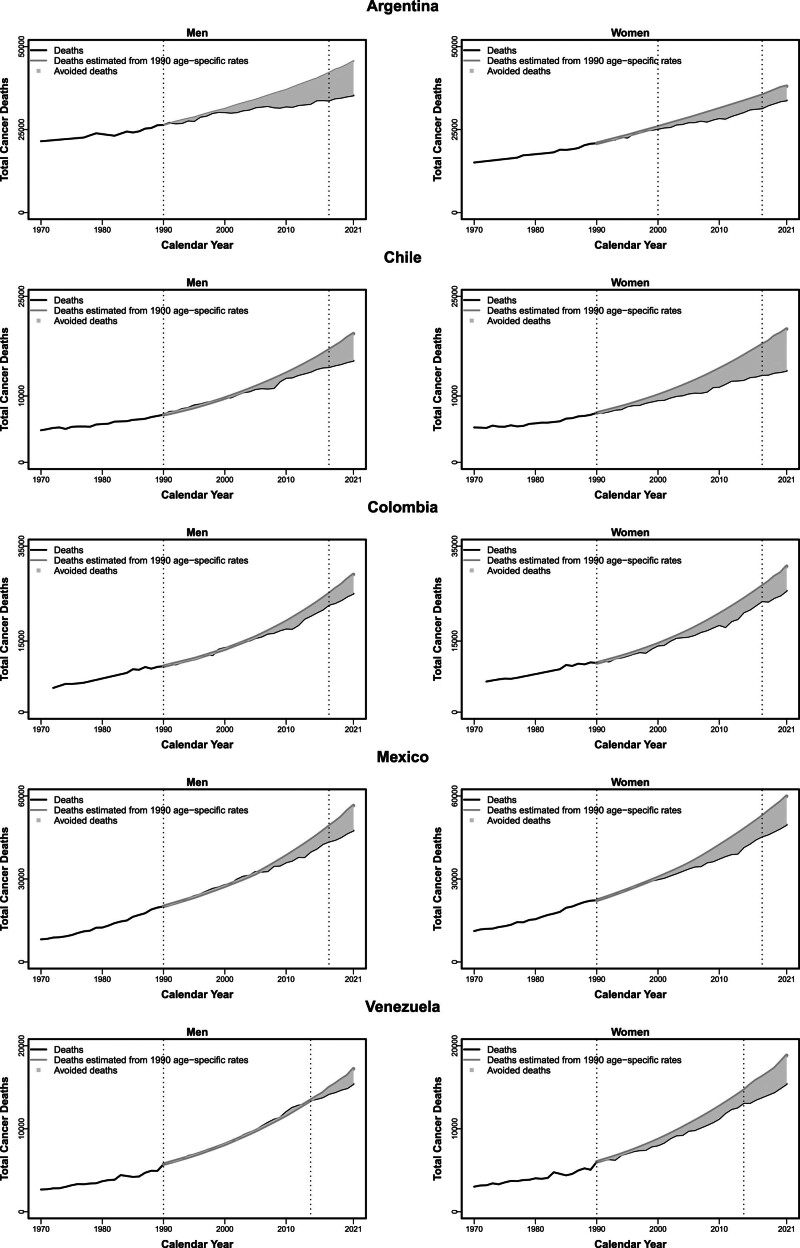
Total avoided cancer deaths for five of the seven Latin American countries considered, in both sexes between the highest rate in 1990 and 2021 (light grey area); observed numbers of cancer deaths from 1990 to 2017 and predicted cancer deaths from 2018 to 2021 (black line); estimated numbers of total cancer deaths by applying 1990 age-specific peak mortality rates (dark grey line). During the 31-year period, a total of about 584 600 cancer deaths have been avoided in five of the seven countries considered (251 300 in men and 333 300 in women). No reduction in cancer deaths was registered in Brazil, Cuba and Venezuelan men. In 2021 alone, about 27 500 deaths are predicted to be avoided in men, but none in Brazil, Cuba and Venezuela, and about 29 600 in women, but not in Brazil and Cuba.

Figure 4 shows the estimated number of avoided cancer deaths in men and women between 1991 and 2021, assuming constant age-specific rates in 1990 (light grey area). Over the 31-year period, we estimated a substantial amount of avoided deaths in most of the major Latin American countries: in Argentina a total of over 180 000 total cancer deaths were avoided (122 700 in men and 59 400 in women), in Chile 99 600 deaths (30 000 in men and 69 600 in women), in Colombia 92 200 deaths (34 600 in men and 57 600 in women), in Mexico 173 100 deaths (64 000 in men and 109 000 in women), and in Venezuelan women 37 700 deaths. No appreciable reductions in cancer deaths were observed in Brazil, Cuba and Venezuelan men.

## Discussion

Overall, declines in mortality rates from all neoplasms and most major cancer sites in Latin American countries are predicted to continue up to 2021, as reported in other areas of the world (Carioli *et al.*, 2021; Siegel *et al.*, 2021). A few major exceptions to the general favourable pattern were mostly among women: stable rates from all cancers combined and lung cancer in Argentina, colorectal cancer in Brazil and pancreatic cancer in Cuba. Increasing rates were predicted for bladder cancer in Argentinian women, and ovarian cancer in Cuba. However, across the seven selected Latin American countries, there was still a substantial variation in rates, with an around two-fold difference in men and an over 50% difference in women between the highest rates in Cuba and the lowest ones in Mexico. However, the number of cancer deaths was still increasing, due to population growth and ageing.

## Colorectal cancer

Colorectal cancer rates in Latin American countries were lower as compared to the EU and the USA (Carioli *et al.*, 2021; Siegel *et al.*, 2021), particularly in men, apart for Argentina, which showed, instead, relatively high rates and this is consistent with its socioeconomic and cultural characteristics close to those of Europe (Muzzio *et al.*, 2018). Along with socioeconomic development, dietary patterns similar to European ones are widespread in Argentina with a particularly high consumption of red meat (Navarro *et al.*, 2003; Siegel *et al.*, 2019).

Among other major aetiological factors for this cancer, there are overweight, obesity and a sedentary lifestyle. However, despite high prevalence of overweight and obesity in Latin America (Stern *et al.*, 2019), colorectal cancer rates in most countries were relatively low. Physical activity and intake of dietary fibres could have somewhat counterbalanced the unfavourable effects of diet and obesity (Siegel *et al.*, 2019), particularly in Mexico, which showed very low rates (Buamden, 2018). Further, improvements in early diagnosis and treatments may also have contributed to the low mortality rates. Historically low tobacco smoking in these countries may have played some role, too. Differences in the aforementioned risk factors trends and in access to health services could explain the high variability in rates across the considered Latin American countries (Siegel *et al.*, 2017).

As for other areas worldwide (Siegel *et al.*, 2017; Santucci *et al.*, 2021), mortality rates for colorectal cancer in young age groups (under 50 years) in some Latin American countries lacked the favourable predicted patterns. Obesity and alcohol were found to be associated with an excess risk of early onset colorectal cancer (Rosato *et al.*, 2013; Siegel *et al.*, 2019).Thus, these rises in mortality suggest possible changes in risk factors patterns and exposures in most recent generations, and need to be monitored in the future, and preventive strategies implemented, including organized colorectal cancer screening. The American Cancer Association suggested to start colorectal cancer screening at 45 instead of 50 years of age (Santucci *et al.*, 2021).

## Lung and other tobacco-related cancers

The historical low level of smoking in Latin America (Raez *et al.*, 2018) is the likely cause of the lower lung cancer rates as compared to other countries worldwide (Hashim *et al.*, 2016; Carioli *et al.*, 2021). Latin American populations reported low-intensity daily smoking (on average less than 10 cigarettes per day vs. 15–20 per day in high-income countries) and higher smoking cessation rates. This is particularly evident in Mexico, which had the lowest lung cancer rates (Thomson *et al.*, 2021). The prevalence of current cigarette smokers (heavy and passive smokers, as well) decreased among Brazilian adults since 2006, with some slowdown in decreases over more recent periods (Maia *et al.*, 2021). Conversely, Cuba showed exceedingly high rates (particularly in women, as compared to their European and American counterparts) due to its historical high smoking prevalence (Pinheiro *et al.*, 2017). Overall, men experienced greater declines in predicted lung cancer rates as compared to women, and their rates started to decline earlier, reflecting different smoking cohort patterns in men and women. However, lung cancer remained the leading cause of cancer mortality in these countries, suggesting the need to intensify tobacco control programs (Prado-Galbarro *et al.*, 2020) and to improve the access to diagnosis and treatment (Raez *et al.*, 2018).

Although continuous falls are predicted, stomach cancer rates remained comparatively high, particularly in Chile and Colombia. In Latin American countries, *Helicobacter pylori* infection prevalence is still high (Tsang *et al.*, 2021). Among other risk factors, besides tobacco, poor dietary habits (including high consumption of meat, preserved meat and salt), chili pepper consumption and poor food conservation likely played some role (Ruíz-García *et al.*, 2018).

Compared to the EU and North America (Carioli *et al.*, 2021; Siegel *et al.*, 2021), pancreatic cancer rates in Latin American countries remained relatively low. Diabetes and obesity are recognized etiologic factors for this cancer; lower smoking in Latin America contributed to this pattern (Wong *et al.*, 2017). However, this cancer is difficult to diagnose, and caution is needed in results interpretation, since under-estimation and under-certification are possible in some countries.

Again, the more favourable smoking patterns in Latin America may explain the lower bladder cancer rates as compared to other geographical areas. Differences in rates between the two sexes may also reflect different occupational exposures. The presence of arsenic in drinking water in selected areas of Chile and Argentina has been found to be associated to this tumour (Khan *et al.*, 2020).

## Other cancer sites

Breast cancer rates in Latin America were favourable and lower than in the EU and the USA (Carioli *et al.*, 2021; Siegel *et al.*, 2021), except for Argentina, which has a high proportion of European immigrants. Besides improvements in disease diagnosis and treatment, the lower rates may be due to reproductive patterns in these countries (Romieu *et al.*, 2018).

Despite falls in rates for uterine cancer in all analysed countries, mortality for this neoplasm remained particularly high. From WHO official deaths certification data, we are not able to distinguish between cervical and endometrial cancer, but the high observed and predicted rates are likely driven by high cervical cancer incidence registered in this area, because cervical cancer represents the main type of uterine cancer in this region (Torres-Roman *et al.*, 2021). The lack of organized screening programs for early diagnosis and the delay in Pap smear tests and more human papilloma virus (HPV) testing along with higher HPV infection rates, probably caused the higher rates as compared to other countries worldwide. In the future, greater coverage of HPV vaccinations is needed to reduce the rates (Wojtyla *et al.*, 2020).

Ovarian cancer rates were low and favourable in all countries, except for Cuba. Among factors that likely have influenced the favourable rates, there was the use of oral contraceptives, contrasting the effect of a high prevalence of obesity (Stern *et al.*, 2019).

Prostate cancer rates were higher compared to the EU and the USA (Carioli *et al.*, 2021; Siegel *et al.*, 2021). The exceedingly high rates estimated in Cuba were probably related to the large presence of Blacks, characterized by a higher incidence and mortality for this cancer (more advanced and aggressive disease at younger ages) (Chinea *et al.*, 2017). However, predicted trends were favourable in all countries, indicating the efficacy of improved therapies together with some possible impacts of earlier diagnosis (Reis *et al.*, 2020).

Improved management also played a role in the declining patterns in leukaemias in Latin America, although delays in adoption of innovative treatments and disparities in access to healthcare were registered, and hence rates remained higher than in most high income countries of the world (Chiattone *et al.*, 2020).

Predicted estimates should be interpreted with care holding the limitations of the models in mind; in particular, this model is not suited to detect very recent changes in trends or major long term cohort effects. However, the analysis is limited to highly populous countries and this should reduce issues of excessive random variation. Since observed cancer deaths for 2017 are available now in the WHO database, we compared observed cancer rates for 2017 with our previous predictions for that year (Carioli *et al.*, 2017) and we found that for all cancers combined errors in our predictions were less than 5%, except for Colombia and Mexico.

Improvements in cancer management and prevention are needed, particularly in Brazil, Cuba and Venezuelan men, for whom no substantial number of cancer deaths were avoided from 1991. Colombia also showed only small decreases in cancer mortality.

## Acknowledgements

This study was conducted with the contribution of the Italian Association for Cancer Research (AIRC, project N. 22987), MIUR (Ministero dell’Istruzione, dell’Universita` e della Ricerca), with an SIR (Scientific Independence of Young Researchers) 2014 grant (project RBSI1465UH).

Novelty and Impact: Cancer mortality rates in major Latin American countries are predicted to decline until 2021. In Cuba, lung cancer rates remained exceedingly high in both sexes, and in women overtook those for breast cancer. In some countries, colorectal cancer predicted rates under age 50 were unfavourable. Estimated avoided cancer deaths in 1991–2021 are around 585 000 in the selected Latin American countries, but no decline was observed in Brazil, Cuba and Venezuelan men.

### Conflicts of interest

There are no conflicts of interest.
